# Adoption of Decentralization: Are Our Perceptions Holding Us Back?

**DOI:** 10.1007/s43441-024-00636-3

**Published:** 2024-03-28

**Authors:** Lindsay Kehoe, Sara Bristol Calvert, Zachary Hallinan, Morgan Hanger

**Affiliations:** Clinical Trials Transformation Initiative, Duke Clinical Research Institute, 300 West Morgan Street, Suite 800, Durham, NC 27701 United States

**Keywords:** Decentralized clinical trials, Quality by design, Participant-centric trials

## Abstract

Incorporating decentralized approaches into clinical trials is a critical innovation with potential implications for improved accessibility and diversity, as well as lower burden for participants and caregivers. As we move forward in a collective effort to modernize clinical trials, we consistently hear of hurdles that interfere with the adoption of decentralized approaches. But are these hurdles really the impediments we think they are? In this commentary, we offer three perceptions that are commonly heard as impediments to the adoption of digital and decentralized clinical trials. Leveraging the Clinical Trial Transformation Initiative’s Digital Health Trial hub of work, interactions with members and regulators, and observations related to adoption, we address those perceptions and note some resources that exist to overcome them. In working through these barriers, we can instill confidence in sponsors and designers to leverage all the clinical trial design tools available to them to advance the use of decentralized approaches.

## Introduction

For decades, only a small percentage of eligible, and generally willing, patients has participated in clinical trials [1–4]. With recent attention on improving equitable access and diverse trial participation [5–8], many across the clinical trials ecosystem are seeking practical approaches to achieve this critical goal [9–12]. Given that 70 percent of potential participants in the United States live more than 2 h away from the nearest study center [13], and nearly 25 percent of trials are discontinued early (often due to recruitment challenges) [14], there is an urgent need to accommodate a participant’s real life as they sacrifice time, wages, and other responsibilities to contribute to the evidence base for new treatments. Decentralization is one promising approach to address these challenges and improve access.

The Clinical Trials Transformation Initiative (CTTI), a public private partnership co-founded by Duke University and the U.S. Food and Drug Administration (FDA), has defined decentralized clinical trials (DCTs) as *trials in which some or all study assessments or visits are conducted at locations other than the investigator site via any or all of the following: tele-visits; mobile or local healthcare providers, including local labs and imaging centers; and home delivery of investigational products.* The use of digital health technologies, such as electronic sensors and computing platforms, are often important elements of decentralized trials [15, 16].

There is widespread support for decentralization, including regulatory reforms and new guidance, operational and trial design resources from public private partnerships [15, 17, 18], and government funding of innovative pilot studies, infrastructure needs, and sustainable business models [19–21]. The U.S. Congress recently authorized additional financial investment in health through the Advanced Research Projects Agency for Health, which has launched The Advancing Clinical Trial Readiness Initiative with a goal of enabling 90% of all eligible Americans to take part in a clinical trial within a half hour of their home: decentralization—particularly the technologies and infrastructure that will facilitate it—is one of their five focal areas [22].

Despite this momentum, real and perceived challenges remain, limiting wider implementation of decentralized elements. In this commentary the authors, all CTTI staff, describe three commonly stated barriers, comment on the extent to which perceived barriers are manageable, and discuss resources that exist to address them. Our purpose is to instill confidence in sponsors and trial designers to leverage the tools available to them to advance the use of decentralized approaches.

## Overcoming Perceptions

### Perception #1: Flexibility is a Threat to Data Quality

Decentralized trials often provide options to participants for where to undergo study visits and assessments. Though more work is needed to understand how decentralized designs can improve participation and participant diversity [23], small changes in trial design can be meaningful and increase access and/or retention [24]. However, concerns are often raised that such variability and flexibility can create risks to data quality [25].

It is true that “real world” factors can regularly be controlled for when on site: distractions are often less likely, technology issues or missing data can be more readily addressed, procedures are more consistent. It is also true that different sources of data may require integration.

The perception that flexibility is a threat to data quality has grounds for truth if the appropriate planning is not in place. But with proactive training, data management and oversight plans, most risks can be mitigated. CTTI’s Digital Health Trials Hub, which is a collection of resources developed from a series of evidence-driven, multi-stakeholder projects, has recommendations to help manage data and plan decentralized clinical trials [26]. (Table 1).

**Table 1:** Key points from CTTI’s planning decentralized trials and managing data recommendations

Planning decentralized trials	Managing data in digital health trials
· Follow a Quality by Design approach to reduce opportunities for errors that have a meaningful impact on the safety of trial participants or the credibility of the results · Assess which potential study activities are feasible and suitable to conduct remotely · Create proactive oversight plans · Monitor the consistency and comparability of data collection · Evaluate and address risks to the study participants’ privacy and confidentiality · Clearly define responsibilities for triaging and evaluating data to ensure adequate protection of participant safety (according to the requirements in each planned study country) · Plan how to handle system failures at any level	· Quality by Design principles should drive decisions about the quantity of data to be collected · Optimize data accessibility while preventing data access from unauthorized users · Minimize missing data · Monitor data quality centrally through automated processes · Plan appropriately for the statistical analysis of data captured · Conduct small-scale feasibility studies prior to finalizing the protocol design · Identify ways to return value to participants throughout the trial · Apply an end-to-end, risk-based approach to data security · Let data sharing decisions be driven by safety and trial integrity

### Perception #2. Decentralization Undermines Traditional Relationships and Accountability Structures

Previous work has highlighted concerns around roles and responsibilities in decentralized trials [27, 28]. As the field gains more experience, we continue to hear concerns about the relationship between investigator and patient, the overall role of the investigator, and the complexities for sites as they manage an increasing number of technologies and navigate oversight of home health providers.

It can be a conceptual adjustment for a clinician accustomed to meeting with patients face-to-face: how does that responsibility change when trial participants are recruited and “seen” digitally? Some worry about the erosion of these relationships. Looking forward, some evidence indicates that clinicians and investigators are becoming more comfortable with various types of technologies leveraged in DCTs [29]. Additionally, some DCTs are actively exploring ways to leverage technology to strengthen investigator-participant relationships, for instance through optional “town halls” [30].

It is also important to note that decentralization rarely means a trial without sites: sites remain partners who make decentralization possible. When used with appropriate planning, technology for DCTs should support the connection between participants and sites. For example, short virtual check-ins and collection of endpoints using wearables can reduce the number of assessments required at in-person study visits, allowing clinician-investigators to focus on required physical assessments, treatments, assessment of well-being, and building the personal connections that many site staff and patients value.

Challenges also remain around defining what constitutes a site in the context of some innovative trial designs, whether the level of oversight is clear, and who is accountable for the safety and welfare of the participant. In response, professional organizations and regulators are converging to address the issue. For example, American Society of Clinical Oncology’s recent call to action to advance patient-focused and decentralized clinical trials focuses on three key strategies: “confront challenges with the interpretation of [the Food and Drug Administration] FDA’s Form 1572 requirements (Statement of Investigator); broaden acceptance of local laboratories and imaging centers; and invest in the creation of effective, sustainable partnerships between research centers and local providers” [31].

CTTI has also developed recommendations for supporting sites to facilitate effective site-participant communication and help build site DCT infrastructure [32] (Table 2).

**Table 2:** Key points from CTTI’s supporting sites recommendations

· Educate sites about the anticipated benefits and challenges of DCT elements and digital health technologies · Listen carefully to the experiences, perceptions, and recommendations of investigators and site personnel, ensuring effective two-way communication · Assess the additional time and cost that will be required for sites to implement any DCT and digital health elements · Clearly delineate responsibilities and consider implementing alternative payment structures in contracts · Agree on expectations and processes for the oversight of any non-site trial personnel, such as mobile nurses
· Ensure sites have the appropriate infrastructure to support the planned DCT elements · Confirm appropriate plans and policies are in place to handle technology issues, malfunctions, and failures that could result in risks to participant safety and/or data loss · Develop effective training modules for trial personnel (both site-based and mobile) around planned DCT elements and digital health technologies · Support Effective Site/Patient Communication

Moving forward, we should continue to aim to empower patients, leverage technology to improve provider-participant interactions and trust, and increasingly incorporate DCT elements that offer flexibility for participants when it makes sense to do so.

### Perception #3: Regulators Haven’t Made it Clear What We Can Do

Stakeholders frequently express that that lack of clear regulations are a major impediment to widespread use of DCT elements. That said, regulators encourage “modernizing the design and conduct of clinical trials, making them more agile without compromising data integrity or participant protections” [33], and the FDA has taken strides to address these challenges in several ways. For example, the agency has offered guidance that supports decentralized approaches, including the 2023 draft guidance *Decentralized Clinical Trials for Drugs, Biological Products, and Devices* [34]*.* In October 2023, the FDA, in collaboration with CTTI, also convened a public meeting on Mitigating Clinical Study Disruptions During Disasters and Public Health Emergencies (PHEs) to share, learn from, and build on experiences during the Covid-19 pandemic, during which many trials were able to move forward in novel ways [35].

It is essential to recognize that regulations cannot account for every option and contingency. Rather, sponsors can leverage the principles of the guidance and apply them to their situation. Trial designers are also encouraged to engage early with the relevant health authorities when considering new approaches. CTTI’s recommendations for Interacting with Regulators can guide conversation during the design and conduct of DCTs [36]. Supporting resources also provide information about data processes to include in submissions to the FDA [37], data that should be made available to inspectors [38], and a guide for engaging with the FDA and/or the European Medicines Agency when developing a digitally derived endpoint [39].

## Conclusion

Despite the long arc of adoption for technologies and capabilities supporting clinical trial execution [40], the COVID-19 pandemic showed us that trials using decentralized approaches can be successfully implemented on a large scale, rapidly, while maintaining participant safety and data integrity. As we increasingly work to make “patients as partners” a reality in medicine, it is critical to both support and empower patients in their trial participation—and decentralized approaches are an increasingly valuable tool for doing so.

Decentralized approaches are not all or none, and their use should be fit for purpose. Only the appropriate DCT elements should be incorporated to ensure that data quality is not compromised and technology is used to help and not hurt. Designers must continue to consider target population and ease of use. Ongoing experimentation along these lines will help create an evidence base around the trial characteristics that encourage broader participation, including from more diverse and historically under-represented populations.

As a public–private partnership, CTTI encourages stakeholders to share their hesitations and their experiences with each other and with regulators. Harnessing our collective experiences—learning from successes and failures—can increase comfort with new methods. Change is constant. Barriers will arise. In order to foster a clinical trials ecosystem that we all want to participate in, we must continue to the leverage resources that exist and work collectively to improve it.

## Data Availability

Not applicable.
